# Antiviral RNAi Response against the Insect-Specific Agua Salud Alphavirus

**DOI:** 10.1128/msphere.01003-21

**Published:** 2022-02-16

**Authors:** Mine Altinli, Mayke Leggewie, Marlis Badusche, Rashwita Gyanwali, Christina Scherer, Jonny Schulze, Vattipally B. Sreenu, Marvin Fegebank, Bernhard Zibrat, Janina Fuss, Sandra Junglen, Esther Schnettler

**Affiliations:** a Bernhard-Nocht-Institute for Tropical Medicine, Hamburg, Germany; b German Centre for Infection Research (DZIF), Partner Site Hamburg-Luebeck-Borstel-Riems, Hamburg, Germany; c MRC-University of Glasgow-Centre for Virus Research, Glasgow, United Kingdom; d Institute of Clinical Molecular Biology (IKMB), Kiel University, Kiel, Germany; e Institute of Virology, Charité-Universitätsmedizin Berlin, Berlin, Germany; f University of Hamburg, Faculty of Mathematics, Informatics and Natural Sciences, Hamburg, Germany; Stanford University School of Medicine

**Keywords:** ASALV, alphavirus, antiviral RNAi, arbovirus, insect-specific virus, mosquito, viral small RNAs

## Abstract

Arboviruses transmitted by mosquitoes are responsible for the death of millions of people each year. In addition to arboviruses, many insect-specific viruses (ISVs) have been discovered in mosquitoes in the last decade. ISVs, in contrast to arboviruses transmitted by mosquitoes to vertebrates, cannot replicate in vertebrate cells even when they are evolutionarily closely related to arboviruses. The alphavirus genus includes many arboviruses, although only a few ISVs have been discovered from this genus so far. Here, we investigate the interactions of a recently isolated insect-specific alphavirus, Agua Salud alphavirus (ASALV), with its mosquito host. RNA interference (RNAi) is one of the essential antiviral responses against arboviruses, although there is little knowledge on the interactions of RNAi with ISVs. Through the knockdown of transcripts of the different key RNAi pathway (small interfering RNA [siRNA], microRNA [miRNA], and P-element-induced wimpy testis [PIWI]-interacting RNA [piRNA]) proteins, we show the antiviral role of *Ago2* (siRNA), *Ago1* (miRNA), and *Piwi4* proteins against ASALV in Aedes aegypti-derived cells. ASALV replication was increased in *Dicer2* and *Ago2* knockout cells, confirming the antiviral role of the siRNA pathway. In infected cells, mainly ASALV-specific siRNAs are produced, while piRNA-like small RNAs, with the characteristic nucleotide bias resulting from ping-pong amplification, are produced only in *Dicer2* knockout cells. Taken together, ASALV interactions with the mosquito RNAi response differ from those of arthropod-borne alphaviruses in some aspects, although they also share some commonalities. Further research is needed to understand whether the identified differences can be generalized to other insect-specific alphaviruses.

**IMPORTANCE** Mosquitoes are efficient vectors for many arboviruses that cause emergent infectious diseases in humans. Many insect-specific viruses (ISVs) that can infect mosquitoes but cannot infect vertebrates have been discovered in the last decade. ISVs have attracted great attention due to their potential use in mosquito or arbovirus control, by either decreasing mosquito fitness or restricting arbovirus replication and transmission to humans. However, ISV-mosquito interactions are not well understood. RNA interference (RNAi) is the most important innate immune response against many arboviruses, while it is unknown if it is antiviral against ISVs. Here, we investigate in detail the antiviral effect of the RNAi response in mosquitoes against an ISV for the first time. Using a recently isolated insect-specific alphavirus, we show that the regulation of virus replication was different from that for arthropod-borne alphaviruses despite some similarities. The differences in mosquito-virus interactions could drive the different transmission modes, which could eventually drive the evolution of arboviruses. Hence, an understanding of mosquito-ISV interactions can shed light on the ecology and evolution of both ISVs and the medically important arboviruses.

## INTRODUCTION

Mosquitoes are efficient vectors for many medically important arthropod-borne viruses (arboviruses) from several RNA virus families such as *Flaviviridae*, *Togaviridae*, *Bunyavirales*, *Reoviridae*, and *Rhabdoviridae* (1). Arboviruses have a complex life cycle consisting of replication in both vertebrate and invertebrate hosts. In the last decade, many viruses that are restricted to invertebrate hosts (i.e., that cannot replicate in vertebrate hosts) have also been discovered (2). These viruses, generally termed insect-specific viruses (ISVs), have been discovered from all major arbovirus families. They are considered promising for many applications, from vaccine development to arbovirus transmission control tools (3). Nevertheless, our knowledge of many important aspects of the biology of ISVs is limited, such as their interactions with the vector species that they infect (4).

Arboviruses establish asymptomatic persistent infections in mosquito vectors, which are attributed to the efficiency of the mosquito innate immune system. As a part of the mosquito innate immune system, RNA interference (RNAi) pathways play a major role in regulating arbovirus infections (5, 6). There are three RNAi pathways in mosquitoes: the microRNA (miRNA), small interfering RNA (siRNA), and P-element-induced wimpy testis (PIWI)-interacting RNA (piRNA) pathways (5, 6). The siRNA pathway is triggered by double-stranded RNA (dsRNA) and categorized as exogenous or endogenous depending on the origin of the dsRNA. Among these, the exogenous siRNA (exo-siRNA) pathway is considered the primary antiviral defense mechanism for mosquitoes and other insects (6, 7). The exo-siRNA pathway can be induced by dsRNA derived from either viral replication or RNA secondary structures, which are cut by *Dicer2* (*Dcr2*) into virus-derived siRNAs (vsiRNA) that are 21 nucleotides (nt) in length (5, 6). These vsiRNAs are then incorporated into the RNA-induced silencing complex (RISC), specifically the *Argonaute2* (*Ago2*) protein, and guide the complex to target complementary viral RNA for subsequent cleavage, resulting in the inhibition of virus replication. vsiRNAs specific to arboviruses are produced during infection by all major arboviruses, proving an interaction with the exo-siRNA pathway (8). Furthermore, the knockdown or knockout (KO) of key players involved in the exo-siRNA pathway, the *Dcr2* and *Ago2* proteins, led to an increase in the replication of all tested arboviruses, supporting the antiviral role of these proteins and the exo-siRNA pathway against arboviruses in mosquitoes (8, 9).

The miRNA pathway is known to regulate the gene expression levels of endogenous transcripts in various organisms, including mosquitoes. The miRNA pathway starts by cleaving primary miRNAs into precursor miRNA (pre-miRNA) molecules in the nucleus. After exportation to the cytoplasm, pre-miRNA is cut to miRNA/miRNA* duplexes of 21 to 22 nt in size by *Dicer1*. miRNAs then guide the miRISC (RISC associated with the miRNA pathway), including the *Ago1* protein, to degrade and/or inhibit the translation of (partially) complementary single-stranded RNAs (ssRNAs) (10, 11). However, our knowledge of the antiviral role of the miRNA pathway in mosquito-virus interactions is limited.

Arbovirus-specific piRNAs of 25 to 29 nt in length have also been reported in infected mosquitoes and mosquito-derived cells (12, 13). In Aedes aegypti*-*derived cells, virus-derived piRNA (vpiRNA) biogenesis is *Piwi5*/*6* (depending on the investigated virus) and *Ago3* dependent for Sindbis virus (SINV), chikungunya virus, and dengue virus (DENV) (12, 14, 15). The transcripts are bound by *Ago3* (sense) and *Piwi5/6* (antisense) and processed in the ping-pong amplification cycle. The resultant vpiRNAs have a bias for either uridine at position 1 or adenine at position 10 in the antisense and sense sequences, respectively (U1 and A10), and a complementary region of 10 nucleotides (12, 14). In contrast, another Piwi protein, *Piwi4*, does not directly bind vpiRNAs of viral or transposon origin (14) but preferentially binds antisense piRNAs derived from endogenous viral elements (EVEs). These EVEs can be integrated into the mosquito genome during RNA virus infection and act as an “adaptive immune response” combined with the produced vpiRNAs and *Piwi4* (16). The knockdown of *Piwi4* transcripts resulted in increased virus titers, supporting its antiviral role. In contrast, the knockdown of piRNA pathway proteins did not have a strong antiviral role against the arboviruses tested so far (13, 17–19), except for Rift Valley fever virus (20). *Piwi4* has been shown to interact with proteins of the piRNA and siRNA pathways; however, its antiviral activity against an arthropod-borne alphavirus is independent of *Dcr2* activity in the Aag2 cells (18).

Compared to arboviruses, ISV interactions with the mosquito RNAi pathways are less studied. Studies on ISVs mainly focused on the detection of virus-specific small RNAs in persistently infected cell lines. Here, the production of vsiRNAs and, in some cases, vpiRNAs was detected for different families, including *Flaviviridae*, *Birnaviridae*, and *Phenuiviridae* (3, 21, 22) Our knowledge on RNAi-ISV interactions is further limited for insect-specific alphaviruses, as no persistently infected cell lines are known (23, 24), and only five insect-specific alphaviruses have been identified in mosquitoes so far: Eilat virus (EILV), Taï Forest alphavirus (TALV), Mwinilunga alphavirus (MWAV), Yada Yada virus, and Agua Salud alphavirus (ASALV) (25–28, 53). So far, only the latter has been studied for its interactions with the RNAi response (28). Indeed, ASALV infection of Aedes albopictus-derived cells induces the production of vsiRNAs but lacks the production of vpiRNAs. Moreover, it is unknown whether the siRNA pathway is antiviral against ASALV.

In addition to the mosquitoes’ ability to control virus replication through the RNAi pathways, viruses can also suppress the RNAi response. Indeed, some ISVs such as Culex Y virus and Mosinovirus are known to interfere with the RNAi response (29–31) by encoding an RNAi suppressor protein. However, it is not known whether this is the case for insect-specific alphaviruses.

Here, we investigated the interactions of ASALV with the mosquito RNAi pathways in detail. We show the antiviral role of the exo-siRNA pathway against ASALV by using A. aegypti-derived *Dcr2* and *Ago2* knockout cell lines. ASALV-specific siRNAs were still produced in the absence of *Ago2* but decreased in the *Dcr2* knockout cell line. ASALV triggered piRNA-like small RNA production through the ping-pong production pathway only in *Dcr2* knockout cells. By knocking down additional key RNAi transcripts, we further show the involvement of *Ago1*, *Ago2*, and *Piwi4* in the antiviral activity against ASALV in A. aegypti-derived cells.

## RESULTS

### ASALV efficiently replicates in AF5 cells.

**FIG 1:**
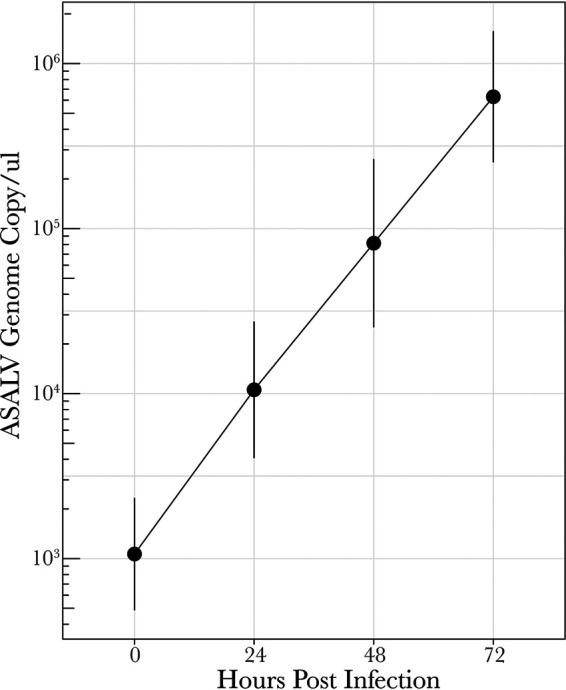
Growth kinetics of ASALV in Aedes aegypti-derived AF5 cells. AF5 cells were infected with ASALV at an MOI of 0.1. The supernatant was collected at different time points (0, 24, 48, and 72 hpi), and ASALV RNA was quantified by qRT-PCR. The averages from three independent replicates (performed in triplicates) are shown with standard errors of the means (SEM).

The successful replication of ASALV was previously shown in A. albopictus-derived C6/36 and U4.4 cells (28). To verify that ASALV could replicate in A. aegypti-derived AF5 cells, a cumulative growth curve was performed by collecting the supernatant every 24 h until 72 h postinfection (hpi). The growth curves showed that ASALV efficiently replicates in AF5 cells (Fig. 1), without any visible cytopathic effect.

### ASALV replication increases in *Dcr2* (AF319) and *Ago2* (AF525) knockout cells.

**FIG 2:**
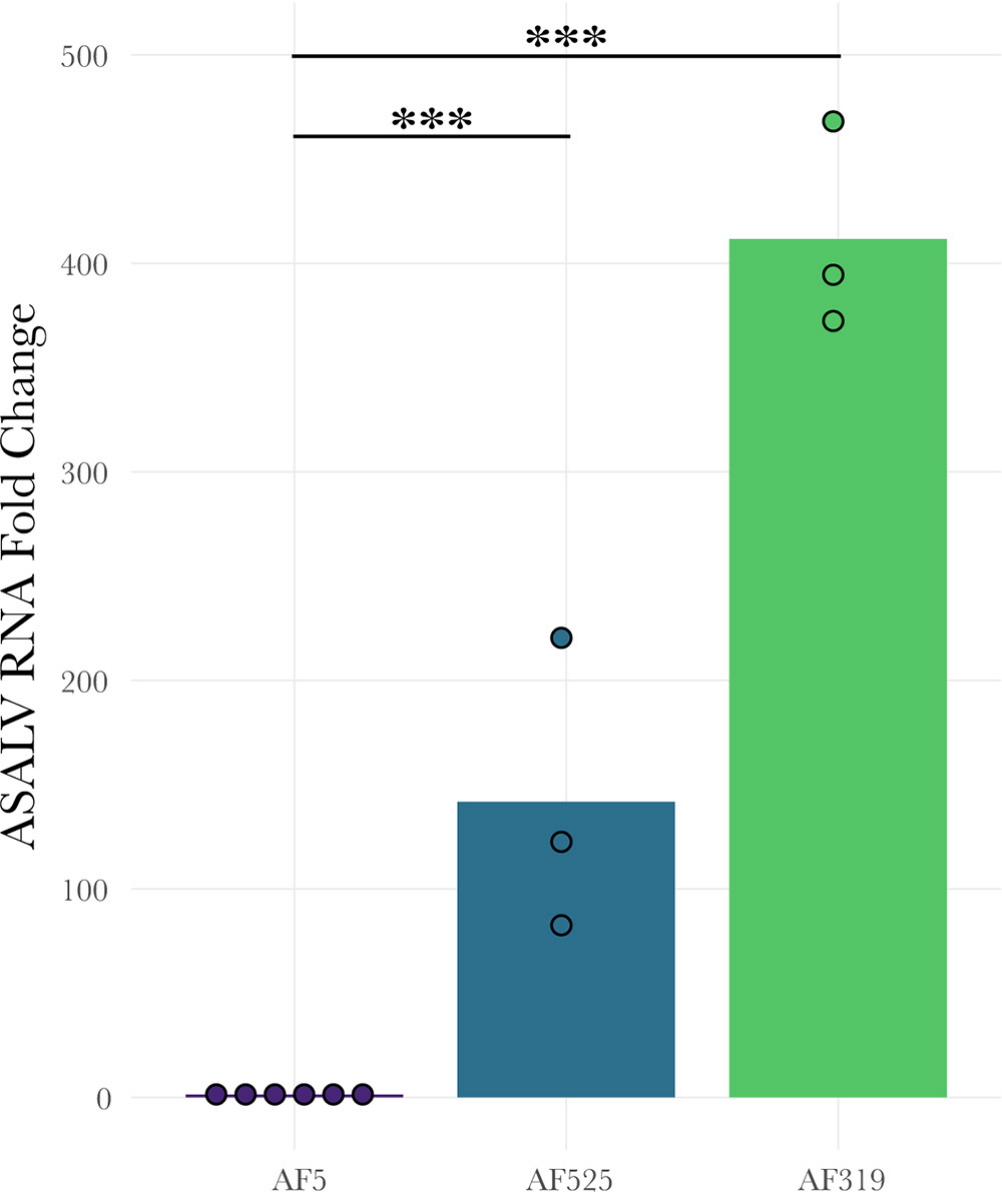
Increased ASALV replication in *Dcr2* (AF319) and *Ago2* (AF525) A. aegypti-derived knockout cells. AF319, AF525, and AF5 cells were infected with ASALV (MOI of 0.5). ASALV RNA fold changes in infected cells were quantified at 48 hpi using the 2^−ΔΔ^*^CT^* method with ribosomal protein S7 RNA as the housekeeping gene and AF5 cells as controls. Three independent replicates were performed for AF525 and AF319 cells (*n* = 3), and AF5 controls were repeated for each group (*n* = 6). Bar plots represent the means from the replicates that were performed (***, *P* < 0.001).

To investigate the effect of the siRNA pathway on ASALV replication, *Ago2* (AF525) and *Dcr2* (AF319) knockout (KO) cells and control AF5 cells were infected with ASALV (multiplicity of infection [MOI] of 0.5). The ASALV RNA fold change in the KO cells compared to AF5 cells at 48 hpi was quantified by quantitative PCR (qPCR). ASALV RNA increased significantly in AF525 (*t* = 5.2385; df = 7; *P* = 0.001) and AF319 (*t* = 21.654; df = 7; *P* < 0.001) cells (Fig. 2) compared to AF5 control cells.

### piRNA-sized small RNAs with ping-pong characteristics are produced only in *Dcr2* KO cells.

**FIG 3:**
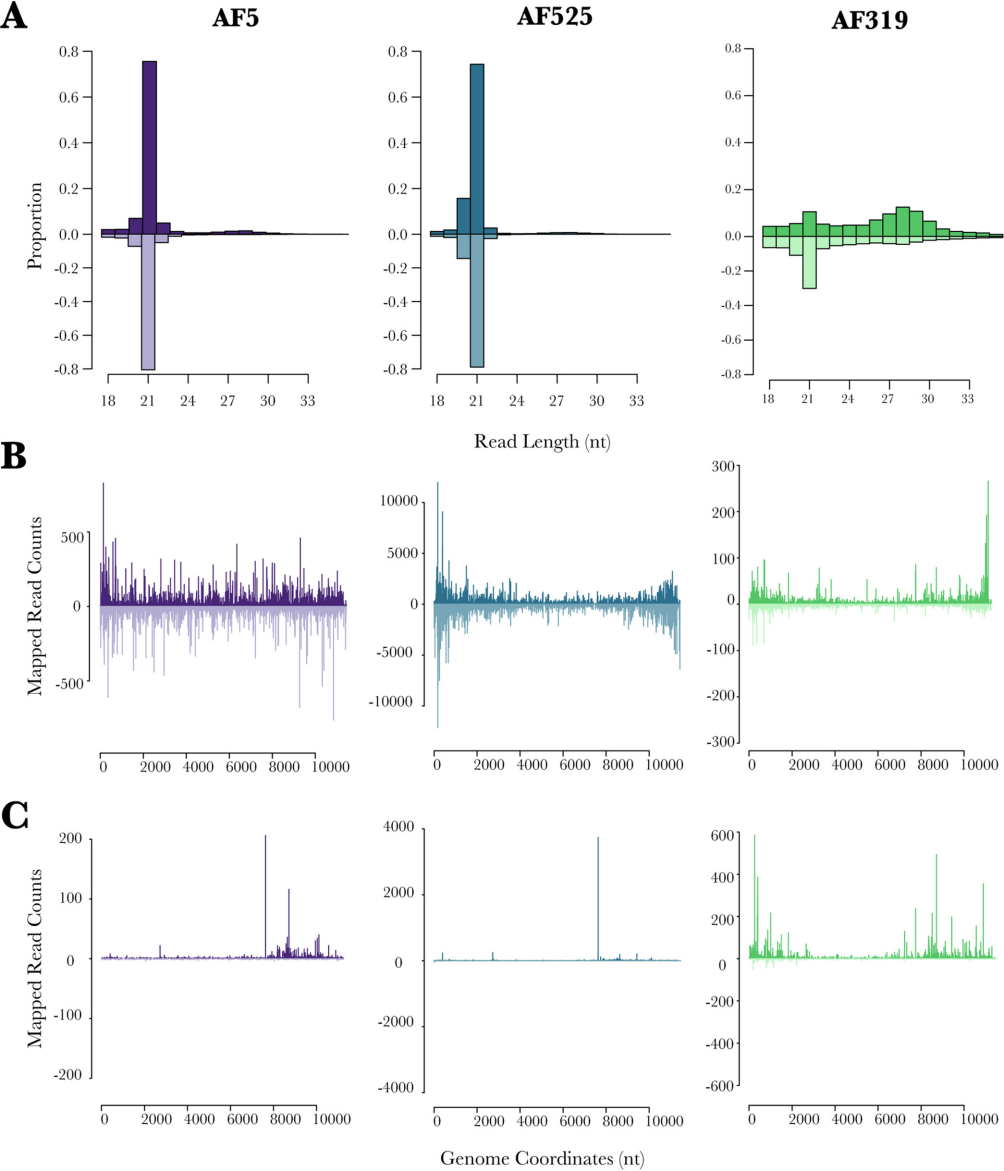
ASALV-specific small RNA production in A. aegypti-derived AF5, AF319 (*Dcr2* KO), and AF525 (*Ago2* KO) cells. Cells were infected with ASALV (MOI of 0.5). Total RNA was isolated from the cells at 48 hpi, and small RNAs (18 to 40 nt) were sequenced and mapped to the ASALV genome (sense) (positive numbers) and antigenome (antisense) (negative numbers). (A) Distribution of the small RNA lengths. The *y* axis shows the proportion of small RNAs of a given length to the total ASALV-specific small RNA reads. (B and C) Mapping of 21-nt (B) and 27-nt (C) small RNAs across the ASALV genome and antigenome. The data shown are representative of results from two independent experiments.

To investigate the production of ASALV-specific small RNAs in the different cells, small RNA sequencing of ASALV-infected cells was performed in A. aegypti-derived AF5, AF319, and AF525 cells. Cells were infected with ASALV (MOI of 0.5), and total RNA was isolated at 48 hpi, followed by small RNA sequencing and bioinformatics analysis. Two independent replicates per cell line were performed, resulting in similar findings (Table 1 and Fig. 3; see also Fig. S1 and S2 in the supplemental material).

**TABLE 1:** Total and ASALV-specific small RNA reads in A. aegypti-derived AF5, AF525 (*Ago2* KO), and AF319 (*Dcr2* KO) cells

Cell line (figure)	Total reads^*a*^			ASALV-specific reads		
	Total no. of reads	No. of 21-nt reads (RPM)	No. of 27–28-nt reads (RPM)	Total no. of reads	Proportion of 21-nt reads to ASALV-specific reads	Proportion of 27–28-nt reads to ASALV-specific reads
AF5 (Fig. 3)	28,193,638	9,996	215	361,122	0.780	0.017
AF5 (Fig. S1)	28,129,604	2,581	113	97,986	0.741	0.033
AF525 (Fig. 3)	22,393,006	147,349	1,308	4,284,285	0.770	0.007
AF525 (Fig. S1)	70,276,976	108,396	490	12,543,700	0.607	0.003
AF319 (Fig. 3)	27,567,645	1,234	1,859	254,288	0.134	0.202
AF319 (Fig. S1)	28,463,344	1,713	2,148	284,136	0.172	0.215

a RPM, reads per million.

10.1128/msphere.01003-21.1 FIG S1 ASALV-specific small RNA production in A. aegypti-derived AF5, AF319 (Dcr2 KO), and AF525 (Ago2 KO) cells. Cells were infected with ASALV (MOI of 0.5). Total RNA was isolated from the cells at 48 hpi, and small RNAs (18 to 40 nt) were sequenced and mapped to the ASALV genome (sense) (positive numbers) and antigenome (antisense) (negative numbers). (A) Distribution of small RNA lengths. The *y* axis shows the proportion of small RNAs of a given length to the total ASALV-specific small RNA reads. (B and C) Mapping of 21-nt (B) and 27-nt (C) small RNAs on the ASALV genome and antigenome. The data shown are representative of results from two repeats. Download FIG S1, TIF file, 1.2 MB. Copyright © 2022 Altinli et al. 2022 Altinli et al. https://creativecommons.org/licenses/by/4.0/ This content is distributed under the terms of the Creative Commons Attribution 4.0 International license.

10.1128/msphere.01003-21.2 FIG S2 Characterization of ASALV-specific 25- to 29-nt-long small RNAs in A. aegypti-derived AF5, AF319 (Dcr2 KO), and AF525 (Ago2 KO) cells. (A) Overlap frequencies of sense and antisense 25- to 29-nt-long small RNAs. (B) Sequence logo plots showing the sequence bias in various positions of 27-nt (representative of vpiRNAs)-long ASALV-specific small RNAs. (Top) Genome; (bottom) antigenome. The data shown are representative of results from two repeats. Download FIG S2, TIF file, 0.9 MB. Copyright © 2022 Altinli et al. 2022 Altinli et al. https://creativecommons.org/licenses/by/4.0/ This content is distributed under the terms of the Creative Commons Attribution 4.0 International license.

In AF5 cells, ASALV-specific siRNAs (Fig. 3A) are produced and map across the genome (sense) and antigenome (antisense) (Fig. 3B), similar to the results previously observed in U4.4 cells (28). Similarly, in *Ago2* KO AF525 cells, the majority of ASALV-specific small RNAs are 21-nt-long vsiRNAs (Fig. 3A). They also map across the whole genome and antigenome although with a bias to the 5′ and 3′ ends (Fig. 3B), which is not observed in AF5 cells. In *Dcr2* KO AF319 cells, ASALV-specific siRNAs are strongly decreased, and a majority of them map to the 3′ end of the ASALV genome.

**FIG 4:**
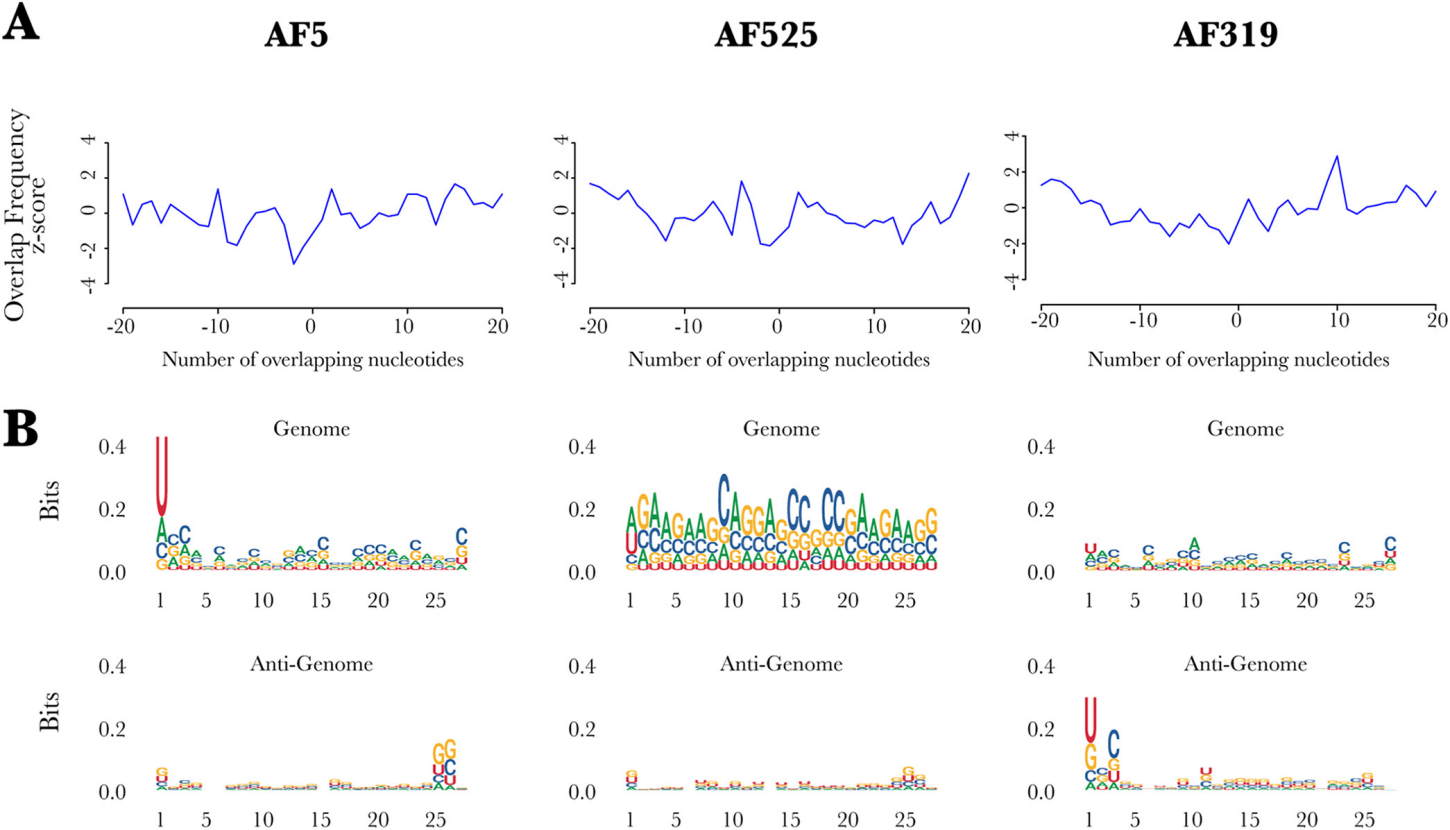
Characterization of ASALV-specific 25- to 29-nt-long small RNAs in A. aegypti-derived AF5, AF319 (*Dcr2* KO), and AF525 (*Ago2* KO) cells. (A) Overlap frequencies of sense and antisense 25- to 29-nt-long ASALV-specific small RNAs. (B) Sequence logo plots showing the sequence bias in various positions of 27-nt (representative of vpiRNAs)-long ASALV-specific small RNAs for genomic (top) and antigenomic (bottom) small RNAs. The data shown are representative of results from two independent experiments.

piRNA-sized small RNAs were observed at a low concentration in both AF5 and AF525 cells (Fig. 3A to C) and did not show the “ping-pong” amplification characteristics (Fig. 4). In contrast, AF319 cells produce ASALV-specific piRNA-sized small RNAs (Fig. 3A to C) with the ping-pong amplification characteristics (Fig. 4). Antisense and sense piRNA-sized small RNAs showed a clear 10-nucleotide overlap. Adenine was the most frequent nucleotide at the 10th position of the sense piRNA-sized small RNA sequence, although the bias was not very strong. In antisense piRNA-sized small RNA sequences, uridine was the most frequent nucleotide at the first position (Fig. 4).

In all cells, piRNA-sized small RNAs were mapped around the subgenomic promoter and 5′ end of the subgenomic RNA, encoding the capsid protein, similar to vpiRNAs produced by arthropod-borne alphaviruses (14, 17). However, in AF319 cells, some piRNA-sized small RNAs also map to the 5′ end of the genome (Fig. 3C).

Taken together, vsiRNAs are the main small RNA species produced against ASALV infection under normal circumstances. In the absence of *Dcr2*, ASALV can induce piRNA-sized small RNAs with sequence characteristics indicative of the ping-pong amplification pathway.

### siRNA pathway, miRNA pathway, and *Piwi4* are involved in the antiviral RNAi response against ASALV.

**FIG 5:**
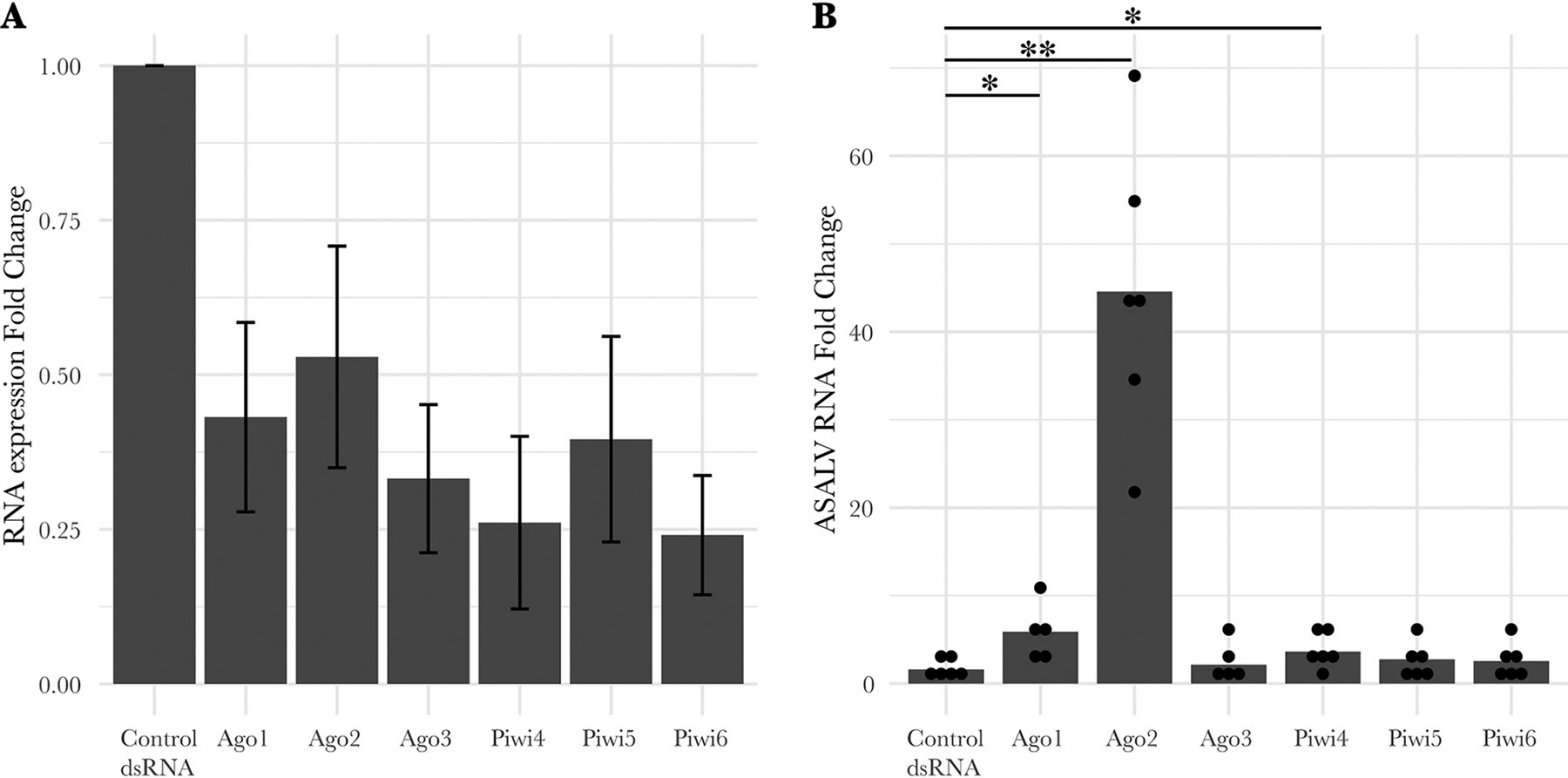
*Ago1*, *Ago2*, and *Piwi4* silencing increases ASALV replication in A. aegypti-derived AF5 cells. Cells were transfected with either gene-specific dsRNAs or control dsRNA (LacZ specific). The following day, cells were infected with ASALV (MOI of 0.5), and total RNA was isolated at 48 h postinfection. (A) mRNA targets were quantified using gene-specific primers and ribosomal protein S7 RNA as the housekeeping transcript. The 2^−ΔΔ^*^CT^* values of mRNA targets were calculated with the mean normalized RNA expression of a given transcript in the control cells, within the same replicate, as a control. The resulting mean fold changes and standard errors of the means are shown. (B) ASALV RNA was quantified using ASALV-specific primers and ribosomal protein S7 RNA as the housekeeping transcript. ASALV RNA fold changes were calculated using the 2^−ΔΔ^*^CT^* method with the mean normalized expression of ASALV RNA, of all replicates, in the control cells as a control. Bar plots represent the mean fold changes calculated for each group. At least five independent replicates were performed (*, *P* < 0.05; **, *P* < 0.01).

The increased ASALV infection in the knockout cell lines supports the involvement of the siRNA pathway in antiviral defense against ASALV. To investigate the involvement of the other RNAi pathway proteins against ASALV in A. aegypti*-*derived AF5 cells, transcripts of different RNAi proteins were silenced by transfecting cells with sequence-specific dsRNAs (*Ago1*, *Ago2*, *Ago3*, *Piwi4*, *Piwi5*, and *Piwi6*) prior to ASALV infection (MOI of 0.5) (Fig. 5A).

Successful silencing was verified (Fig. 5A), and viral RNA was quantified in the cells at 48 hpi and compared to the levels in control cells (transfected with dsRNA specific for LacZ [dsLacZ]). The increase in viral replication was statistically significant in cells where *Ago1* (*t* = 2.817; df = 4.665; *P* = 0.040), *Ago2* (*t* = 6.437; df = 5.039; *P* = 0.001), or *Piwi4* (*t* = 2.628; df = 8.543; *P* = 0.029) transcripts were silenced (Fig. 5B). The ASALV RNA fold change was more pronounced in *Ago2*-silenced cells than in *Ago1*- and *Piwi4*-silenced cells (Table S2). Furthermore, when *Piwi4* silencing was conducted using Piwi4-specific siRNAs instead of dsRNAs, ASALV replication increased in both AF5 and AF319 cells, although this increase was not significant (Fig. S3).

10.1128/msphere.01003-21.3 FIG S3 *Piwi4* knockdown in A. aegypti-derived *Dcr2* KO AF319 cells. AF319 and AF5 cells were transfected with either Piwi4-specific siRNA or control siRNA, followed by ASALV (MOI of 0.5) infection. *Piwi4* transcript levels (A) and ASALV RNA fold changes (B) in infected cells were quantified at 48 hpi, using the 2^−ΔΔ^*^CT^* method with ribosomal protein S7 RNA as a housekeeping gene and control siRNA-transfected cells. Six independent replicates were performed for AF5 and AF319 cells. Bar plots represent the means for the performed replicates (*, *P* < 0.05; **, *P* < 0.01; ns, not significant). Download FIG S3, TIF file, 0.8 MB. Copyright © 2022 Altinli et al. 2022 Altinli et al. https://creativecommons.org/licenses/by/4.0/ This content is distributed under the terms of the Creative Commons Attribution 4.0 International license.

10.1128/msphere.01003-21.6 TABLE S2 ASALV RNA fold changes in knockdown experiments. Values are rounded to 3 decimal points and represent the means from at least 5 replicates. Download Table S2, XLSX file, 0.01 MB. Copyright © 2022 Altinli et al. 2022 Altinli et al. https://creativecommons.org/licenses/by/4.0/ This content is distributed under the terms of the Creative Commons Attribution 4.0 International license.

### No RNAi suppressor effect of ASALV was detected in AF5 cells.

Several insect viruses have been reported to encode proteins that interfere with the antiviral RNAi pathway, named viral suppressors of RNAi (VSRs). VSRs can interfere at different steps of the RNAi pathways by interacting with key molecules (e.g., dsRNA or siRNAs) or proteins (e.g., *Ago2* or *Dcr2*), mostly of the exo-siRNA pathway. To determine if ASALV can suppress the exo-siRNA response in mosquito-derived cells, a previously used luciferase-based RNAi suppressor assay was performed (18, 32). AF5 cells were either infected with ASALV (MOI of 10) or mock infected. After 24 hpi, cells were cotransfected with firefly luciferase (FFluc) and *Renilla* luciferase (internal control) expression constructs as well as dsRNA (FFluc or LacZ as a control) or siRNA (siRNA against FFluc [siFFluc] or against hygromycin B (siHyg) as a control) to induce silencing. Luciferase activity was measured 24 h posttransfection, and the levels of sequence-specific silencing of firefly luciferase in ASALV- and mock-infected cells were compared.

**FIG 6:**
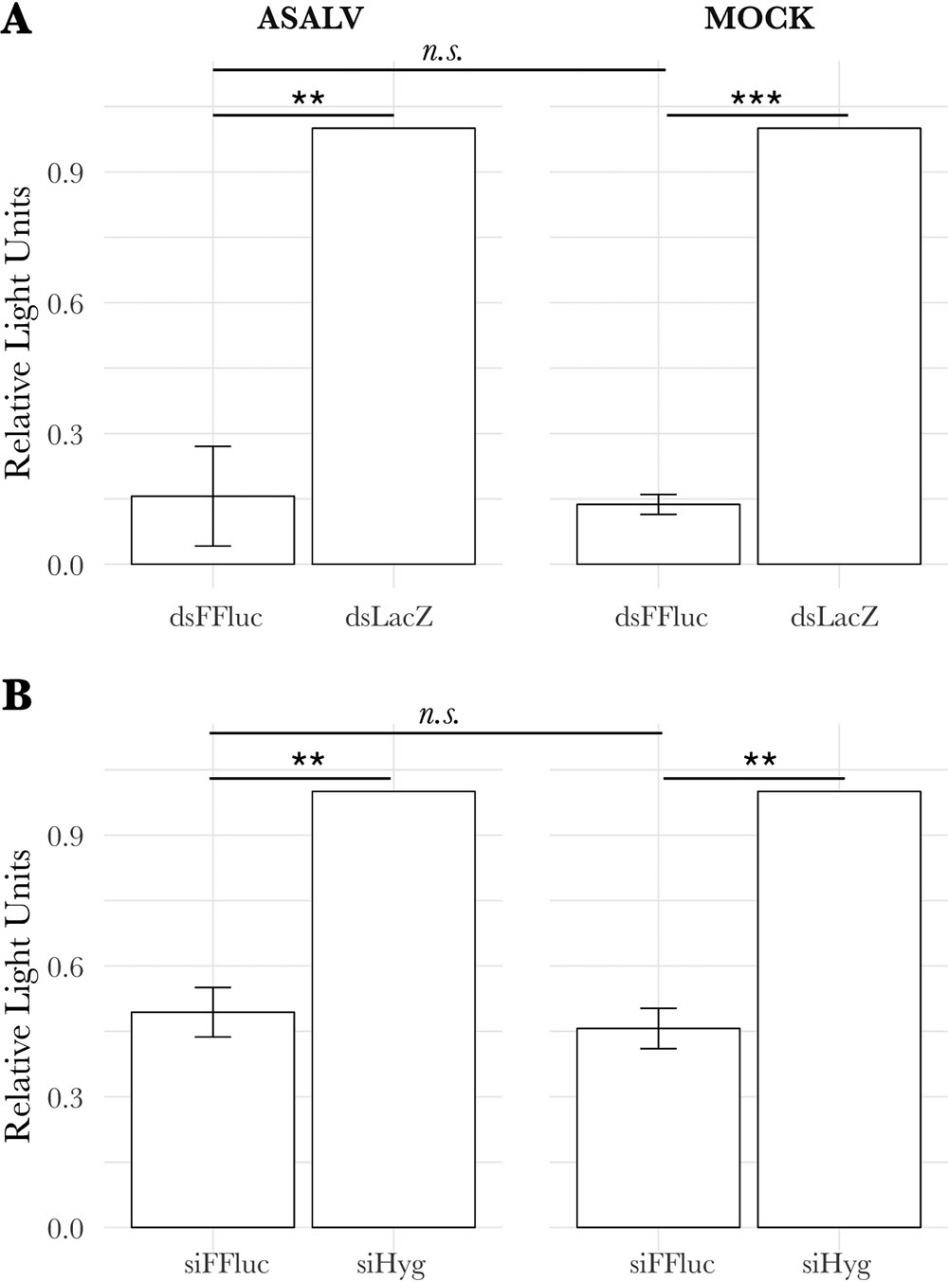
No RNAi suppressor effect of ASALV was detected in AF5 cells. AF5 cells were either mock infected (cell culture medium) or infected with ASALV (MOI of 10). Next, cells were transfected with firefly luciferase (FFluc) and *Renilla* luciferase (*Rluc*) expression constructs and either 0.5 ng dsRNA (A) or 0.1 ng siRNA (B). Luciferase was measured using the dual-luciferase assay, and FFluc expression was normalized to *Rluc* expression as an internal control (relative light units). FFluc/*Rluc* expression levels in the dsRNA (dsFluc)- or siRNA (siFluc)-transfected cells were normalized to those in control transfected cells (dsLacZ or siHyg). The means from three independent experiments in triplicates are shown with SEM (***, *P* < 0.001; **, *P* < 0.01; n.s., not significant).

Relative luciferase activity was significantly reduced in cells transfected with FFluc dsRNA compared to the controls in both ASALV-infected (*t* = −12.785; df = 2; *P* = 0.006) (Fig. 6A) and mock-infected (*t* = −65.212; df = 2; *P* < 0.001) (Fig. 6A) cells. Similarly, luciferase expression was significantly silenced when ASALV-infected (*t* = −15.469; df = 2; *P* = 0.004) or mock-infected (*t* = −20.322; df = 2; *P* = 0.002) cells were transfected with siFFluc compared to control siRNA transfection (Fig. 6B). No difference in the silencing of luciferase could be observed between mock- and ASALV-infected cells regardless of whether the silencing was induced by dsRNA (*t* = 0.281; df = 2.160; *P* = 0.803) (Fig. 6A) or siRNA (*t* = 0.881; df = 4; *P* = 0.428) (Fig. 6B). Hence, in our experimental setting, we did not detect any significant RNAi suppressor activity of ASALV in AF5 cells at either 24 h posttransfection (Fig. 6) or 48 h posttransfection (Fig. S4).

10.1128/msphere.01003-21.4 FIG S4 RNAi suppressor assay in AF5 cells. AF5 cells were seeded into 96-well plates and either infected with ASALV (MOI of 5) or mock infected. At 24 hpi, cells were cotransfected with firefly luciferase (15 ng/well) and *Renilla* luciferase (internal control) (2.5 ng/well) expression constructs as well as dsRNA (FFluc or LacZ as a control) (0.3 ng/well) to induce silencing. Luciferase activity was measured 24 h after transfection and 48 h after transfection, and sequence-specific silencing of firefly luciferase in ASALV- and mock-infected cells were compared. Luciferase was measured using the dual-luciferase assay, and FFluc expression was normalized to that of *Rluc* as an internal control (relative light units). FFluc/*Rluc* expression levels in the dsRNA (dsFluc)-transfected cells were normalized to the levels in the control transfected cells (dsLacZ). The means from three independent experiments in triplicates are shown with SEM (***, *P* < 0.001; n.s., not significant). Download FIG S4, TIF file, 0.3 MB. Copyright © 2022 Altinli et al. 2022 Altinli et al. https://creativecommons.org/licenses/by/4.0/ This content is distributed under the terms of the Creative Commons Attribution 4.0 International license.

## DISCUSSION

RNA interference (RNAi) is an important antiviral response in insects, including mosquitoes. The interaction between the mosquito RNAi pathways and a variety of viruses can be identified by detecting virus-specific small RNAs and increased viral infection in the case of the silencing of key proteins of the different RNAi pathways. RNAi has been shown to act antivirally in mosquitoes against all viruses tested so far, although differences regarding the importance of specific pathways or proteins have been reported (8). Our knowledge about the antiviral RNAi response in mosquitoes comes from arbovirus studies, although mosquitoes often harbor insect-specific viruses (ISVs). Small RNAs specific to a variety of ISVs were found in infected cells and mosquitoes. However, the antiviral role of the RNAi pathway against ISVs is not known (3). Here, we identified the antiviral function of the mosquito RNAi pathways against an insect-specific alphavirus for the first time.

The only previous study investigating an RNAi response specific to an insect-specific alphavirus showed the production of ASALV-specific 21-nt vsiRNAs in *A. albopictus*-derived (U4.4) cells, although no vpiRNAs were observed (28). Our results confirm this previously reported lack of ASALV-specific piRNA production in A. aegypti-derived RNAi-competent AF5 cells (Fig. 4). In contrast, arthropod-borne alphaviruses induce both vsiRNAs and vpiRNAs *in vitro* in A. aegypti- and *A. albopictus*-derived cell lines as well as in mosquitoes (17, 33–37). Despite the difference in the small RNAs that are produced during infection, the mapping of ASALV-specific siRNAs (in both AF5 and U4.4 cells) was very similar to the mapping of arthropod-borne alphaviruses. Both map along the genome and antigenome, more or less equally, with some cold and hot spots (17, 35, 36). This suggests that similar to arthropod-borne alphaviruses, ASALV also mainly induces vsiRNA production through dsRNA replicative intermediates.

ASALV replication is increased in both *Ago2*-silenced (Fig. 5B; see also Table S2 in the supplemental material) and *Ago2* or *Dcr2* knockout (Fig. 2) cells, highlighting the antiviral role of the exo-siRNA pathway against ASALV. Similarly, silencing or knockout of *Ago2* or *Dcr2* induced an increase in infection by the tested arthropod-borne alphaviruses (15, 17, 19, 38, 39). Furthermore, similar results have been found for arboviruses belonging to other virus families or orders (8), except for Zika virus (ZIKV), where no antiviral activity was reported for *Ago2* in silenced cells (9, 19). For the arthropod-borne alphavirus Semliki Forest virus (SFV), the magnitudes of the increase in infection were similar in *Dcr2* and *Ago2* knockout cells (9). In contrast, for ASALV, the differences between *Ago2* and *Dcr2* knockout cells suggest an additional role of *Dcr2* in the antiviral response against ASALV independent of *Ago2*. For instance, *Dcr2* can detect viral RNA and induce an antiviral protein, Vago, which activates the Jak-STAT pathway, leading to an antiviral effect in Culex quinquefasciatus (Hsu)-derived cells (40, 41). Notably, however, Vago does not seem to be induced in infected A. aegypti-derived Aag2 cells (42). Alternatively, this increased antiviral effect of *Dcr2* against ASALV might be linked to another as-yet-unknown antiviral pathway related to *Dcr2* activity.

ASALV-specific piRNA-sized small RNAs with ping-pong amplification characteristics were produced only in *Dcr2* knockout cells. Similar to the results with arthropod-borne alphaviruses, ASALV-specific piRNA-sized small RNAs were mainly produced from the positive (genomic) strand, in contrast to vsiRNAs, which have similar amounts of sense and antisense RNA molecules. A likely explanation for this is the *Dcr2*-dependent production mechanism of vsiRNAs from dsRNA molecules. In contrast, the production of vpiRNAs is Dcr independent and not dependent on dsRNA inducer molecules. Previous reports have also shown an increase of SFV-specific vpiRNAs in cells lacking the *Dcr2* protein (18). It is possible that the increase in vpiRNA production is a result of (i) the increased viral replication due to the lack of the antiviral *Dcr2* protein, (ii) the high concentration of ASALV RNA in the cytoplasm that is not cut into vsiRNAs, or (iii) a combination of both. Although ASALV replication was increased in *Ago2* knockout cells, no ping-pong-specific vpiRNAs were detected. While this could mean that increased viral replication is not solely sufficient for ASALV-specific vpiRNA production, it has to be noted that the increase in ASALV replication in *Ago2* knockout cells was still lower than that in *Dcr2* knockout cells. Therefore, it could be that the increased ASALV RNA concentration in *Ago2* KO cells is not sufficient to trigger vpiRNA production, in contrast to *Dcr2* KO cells. In addition, it is likely that in *Dcr2* knockout cells specifically, the amount of viral dsRNA molecules would increase. As the precise trigger for vpiRNA production in mosquitoes is not yet known, it could be that the concentration of ASALV dsRNA in *Dcr2* knockout cells could play a role in triggering vpiRNA production. On the other hand, the putative essential proteins for the biogenesis of vpiRNAs, *Piwi5* and *Ago3*, were not antiviral against ASALV (Fig. 5B), consistent with findings from arthropod-borne alphaviruses (14, 15, 19).

The silencing of *Piwi4* resulted in a small but statistically significant increase in ASALV replication, as was previously shown for other arboviruses, including alphaviruses (18, 43). The general antiviral role of *Piwi4* is still not clear. *Piwi4* is not required for the production of SFV- or SINV-specific vpiRNAs, but it was recently shown to bind DENV-specific piRNAs derived from viral cDNA in infected A. aegypti (16). While an interaction between *Piwi4* and piRNA as well as siRNA pathway proteins, including *Dcr2*, has previously been shown, *Piwi4* antiviral activity is independent of *Dcr2* in SFV-infected cells (18, 44). To check this for ASALV, we silenced *Piwi4* by siRNAs in both *Dcr2*-competent and *Dcr2* knockout cell lines. While the silencing of *Piwi4* through siRNA increased ASALV replication, the increase was not significant in either of the cell lines (Fig. S3). Whether this is due to the lower silencing efficiency of Piwi4 with siRNAs than with dsRNA is not known. In addition, in the knockout cell lines, it is possible that the high viral replication does not leave much room for additional significant increase in the ASALV replication upon siRNA-based silencing. Hence, it was not possible to conclude whether the effect of *Piwi4* is *Dcr2* independent.

Our results suggest an antiviral effect of *Ago1*, which is primarily involved in the miRNA pathway (Fig. 5). Although the mosquito miRNA response has been shown to interact with viruses through either mosquito- or virus-encoded miRNAs (6), the silencing of *Ago1* has not resulted in changes in arboviral alphavirus replication (17, 45, 46). Similar increases in virus infection upon Ago1 silencing have been reported for midge-borne orthobunyaviruses in A. aegypti-derived cells, in contrast to mosquito-borne orthobunyaviruses (43). Additional experiments are needed to determine if the difference in *Ago1* activity against arthropod-borne alphaviruses compared to insect-specific alphaviruses can be generalized.

Many viruses infecting insects encode proteins to suppress the RNAi pathway, such as flock house virus or Culex Y virus (47). Several arboviruses, such as dengue and West Nile viruses, have also been shown to interfere with the RNAi response by employing competitive substrates for *Dcr2*, derived from their nucleic acids (47). Furthermore, recent work has identified the nonstructural protein NS2A of flaviviruses as a potent suppressor of RNAi (48). In our experimental system, we did not observe any RNAi suppressor activity by ASALV at either 24 or 48 h posttransfection (Fig. S4).

ISVs belonging to some of the arbovirus families and orders, such as *Bunyavirales* (49) and *Flaviviridae* (50), are thought to be ancestral to arboviruses, suggesting that dual-host (invertebrate-vertebrate) tropism evolved from invertebrate-specific viruses. As not many insect-specific alphaviruses have been discovered so far, it is difficult to identify whether ISVs or the arthropod-borne alphaviruses are the ancestors in the alphavirus genus (51). Nevertheless, like other insect-specific alphaviruses so far, ASALV is basal to the Western equine encephalitis virus complex clade, suggesting that arthropod-borne alphaviruses in this clade could have evolved from an ancestral insect-specific virus (28, 51). It is also possible that the changes in the mosquito-virus interactions drive their evolution, resulting in their ability to transmit to vertebrates. In this context, differences between arboviral and insect-specific alphavirus interactions with mosquito RNAi pathways could be one of the reasons why ISVs were restricted to invertebrate hosts. In contrast to the arthropod-borne alphaviruses studied so far, we showed that ASALV-specific vpiRNAs are not produced in *Dcr2*-competent cells, and *Ago1* was antiviral against ASALV. However, to be able to generalize this observation to other insect-specific alphaviruses, more studies describing their interactions with mosquito hosts are needed. Further studies taking both the persistent nature of ISVs and the tissue specificity of the RNAi response into account could determine whether the interactions of insect-specific alphaviruses with the RNAi pathways restrict ISVs to their mosquito hosts.

## MATERIALS AND METHODS

### Cell lines.

Aag2-AF5 (ECACC 19022601) is a single-cell clone of Aedes aegypti-derived Aag2 cells. Aag2-AF319 (ECACC 19022602) is a *Dcr2* knockout (KO) cell line derived from AF5 cells (18), and AF525 is an *Ago2* knockout cell line also derived from AF5 cells (9). Aedes albopictus-derived C6/36 cells were used for virus production.

All cell lines were kept in Leibovitz’s L15 medium (Thermo Fisher Scientific) supplemented with 10% tryptose phosphate broth (Gibco Life Technologies), 10% fetal bovine serum (Thermo Fisher Scientific), and 1% penicillin-streptomycin (Thermo Fisher Scientific). All cell lines were grown at 28°C.

### ASALV stock.

Previously isolated and plaque-purified ASALV was used for all experiments (28). Virus stocks were produced by inoculating C6/36 cells. The supernatant was harvested upon the observation of morphological changes and was cleared from the cell debris by centrifugation. For 50% tissue culture infective dose (TCID_50_) virus quantification, 4 × 10^4^ C6/36 cells per well were seeded into 96-well plates 2 h before infection. Serial dilutions were performed in L15 complete medium.

### dsRNA synthesis.

Primers specific for A. aegypti *Ago1*, *Ago2*, *Ago3*, *Piwi4*, *Piwi5*, and *Piwi6* (17) and LacZ (Aedes-T7-BGal F/R) (52) flanked by T7 RNA polymerase promoter sequences were used to amplify gene-specific fragments. Amplified fragments were validated by Sanger sequencing. PCR products were used for *in vitro* transcription and subsequent column-based purification using the MEGAscript RNAi kit (Thermo Fisher Scientific) according to the manufacturer’s instructions.

### Growth kinetics.

A total of 4 × 10^5^ AF5 cells per well were seeded into 12-well plates a day prior to infection and kept at 28°C overnight. Cells were infected with ASALV at a multiplicity of infection (MOI) of 0.1. After 1 h of incubation, the infectious medium was replaced with 1 mL of fresh L15 medium with supplements. Samples were taken at different time points (0, 24, 48, and 72 h postinfection [hpi]). Infection and negative controls were performed in triplicates, and three independent experiments were performed. The amount of viral RNA in the supernatant was quantified using RNA isolated from supernatant samples with TRIzol LS (Invitrogen) according to the manufacturer’s protocol. A QuantiTect SYBR green quantitative real-time PCR (qRT-PCR) one-step kit (Qiagen) was used to quantify ASALV using previously established primers (28). Samples were run in technical triplicates. An in-run calibrator and an external standard curve were used to perform absolute quantification using a Roche LightCycler 480 II instrument.

### ASALV infection of knockout cells and small RNA sequencing.

A total of 3 × 10^5^ cells/well (AF5, AF525, and AF319) were seeded into 24-well plates and infected with ASALV at an MOI of 0.5 the following day. Total RNA of infected cells was isolated at 48 hpi with TRIzol according to the manufacturer’s protocol, using glycogen as a carrier. A QuantiTect SYBR green qRT-PCR one-step kit (Qiagen) was used to quantify ASALV using previously established primers (28). ASALV RNA fold changes were calculated using the 2^−ΔΔ^*^CT^* method with ribosomal protein S7 RNA as the housekeeping gene and AF5 cells as the control group.

To investigate the production of ASALV-specific small RNAs in AF5, AF525, and AF319 cells, 8 × 10^5^ cells were seeded into a 6-well plate and infected with ASALV (MOI of 1). Total RNA was isolated at 48 hpi with TRIzol (Ambion), according to the manufacturer’s protocol, with glycogen as a carrier. Small RNAs of 1 μg total RNA were sequenced using BGISEQ-500 at BGI Tech (Hong Kong, China) as previously described (9). For one of the AF525 samples (see Fig. S1 in the supplemental material), total RNA was sequenced at IKMB (Kiel, Germany), using 100 ng total RNA for library preparation with the Nextflex small RNA-Seq kit v3 (PerkinElmer Inc., Waltham, MA, USA), followed by library sequencing on one lane of the NovaSeq6000 SP v1.0 platform (2 by 50 bp). Data analyses were performed as previously described (15). The ASALV genome sequence was used as the template (GenBank accession number MK959115).

### Knockdown experiments.

A total of 2.5 × 10^5^ AF5 cells/well were seeded into 24-well plates the day before transfection with 200 ng of gene-specific dsRNAs or control dsRNA (dsLacZ) per well and transfected using 1 μL of Dharmafect2 reagent (GE Dharmacon). For siRNA knockdowns in knockout cells, 20 nM either *Piwi4*-specific siRNAs or control siRNA (Horizon Discovery) was transfected using 2 μL Dharmafect2 reagent (GE Dharmacon), as previously described (18). The following day, ASALV infection (MOI of 0.5) was performed. At 48 hpi, total RNA was isolated from cells using TRIzol (Ambion). cDNA of 1.5 μg RNA was produced using Moloney murine leukemia virus (M-MLV) reverse transcriptase (Promega) and oligo(dT)_15_ primers (Thermo Fisher Scientific) according to the manufacturers’ protocols. SYBR green qRT-PCR for mRNA targets was performed using gene-specific primers (Table S1) and ribosomal protein S7 RNA as the housekeeping gene transcript. Results were analyzed using the 2^−ΔΔ^*^CT^* method with LacZ dsRNA samples as the control. All qPCRs were performed in technical triplicates.

10.1128/msphere.01003-21.5 TABLE S1 qPCR primers used in the study. Download Table S1, DOCX file, 0.02 MB. Copyright © 2022 Altinli et al. 2022 Altinli et al. https://creativecommons.org/licenses/by/4.0/ This content is distributed under the terms of the Creative Commons Attribution 4.0 International license.

### RNA silencing suppressor assay.

To assess whether the presence of ASALV in cells could suppress the RNA silencing response, AF5 cells were seeded into 24-well plates (1.8 × 10^5^ cells/well) 1 day prior to ASALV infection (MOI of 10). The day after infection, cells (ASALV or mock infected) were transfected with firefly and *Renilla* luciferase expression constructs, pIZ-Fluc and pAcIE1-Rluc (18, 32), and either 0.5 ng dsRNA (either dsFluc or dsLacZ as a negative control) or 0.1 ng siRNA (either siFluc or siHyg as a negative control) using 1 μL of Dharmafect2. At 24 h posttransfection, the cells were lysed, and luciferase was measured with a dual-luciferase assay (dual-luciferase reporter assay system; Promega) according to the manufacturer’s protocol on a Glomax luminometer.

### Statistical analyses.

R (version 3.5.2) was used for statistical analyses. First, the normality (Shapiro-Wilk test) and variance (*F* test) of the data were tested. Student’s *t* test was used for normally distributed homoscedastic data, or the Welch *t* test was used for normally distributed heteroscedastic data. A *P* value of <0.05 was considered statistically significant.

### Data availability.

Small RNA sequencing data are available in the NCBI Sequence Read Archive under BioProject accession number PRJNA725665.
