# Supplementary figures and images for: Antiviral RNAi Response against the Insect-Specific Agua Salud Alphavirus
Source: mSphere. 2022 Feb 16;7(1):e01003-21. doi: 10.1128/msphere.01003-21 (PMC8849343; doi:10.1128/msphere.01003-21)

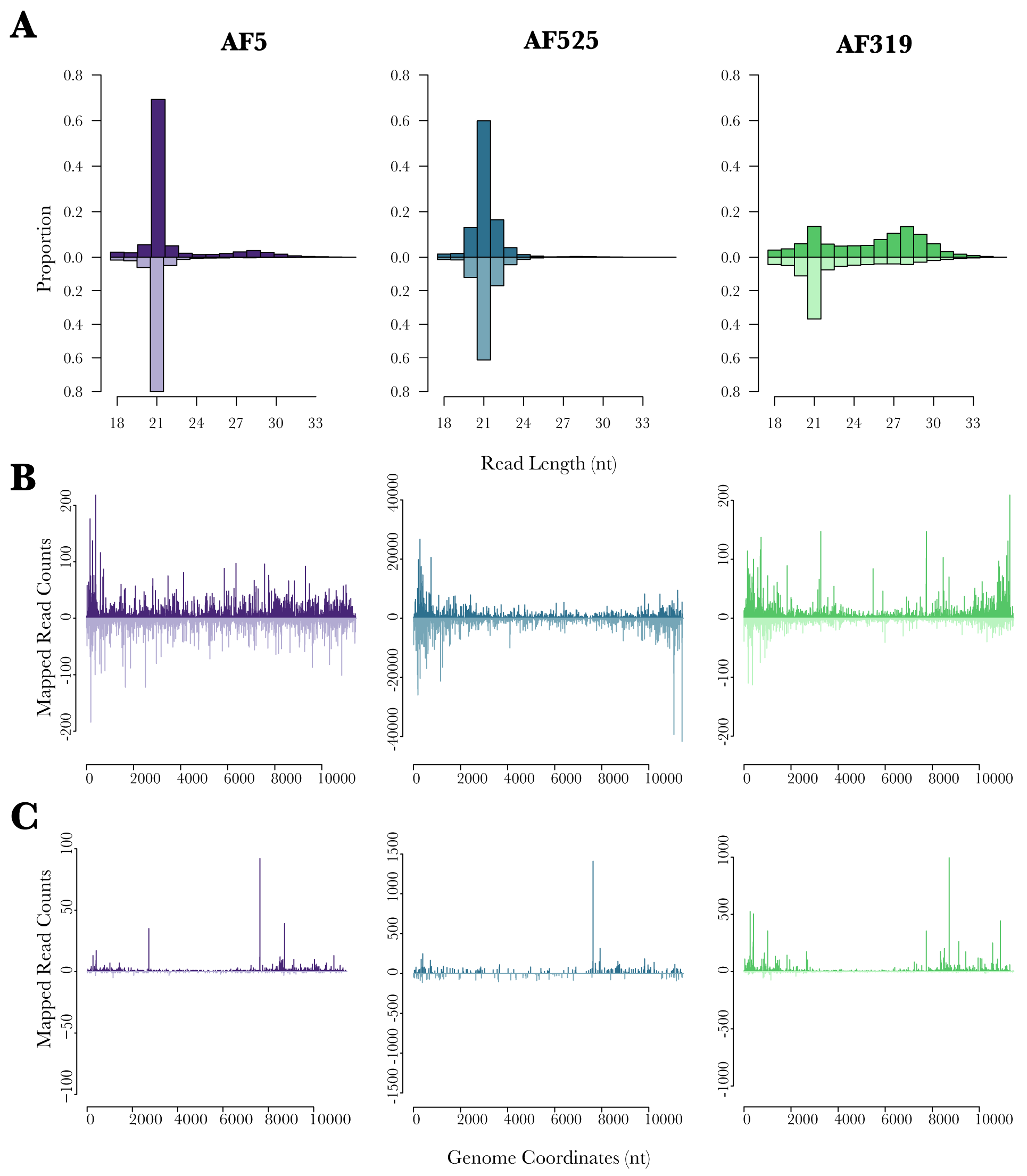

Supplement: FIG S1 [file msphere.01003-21-sf001.tif]

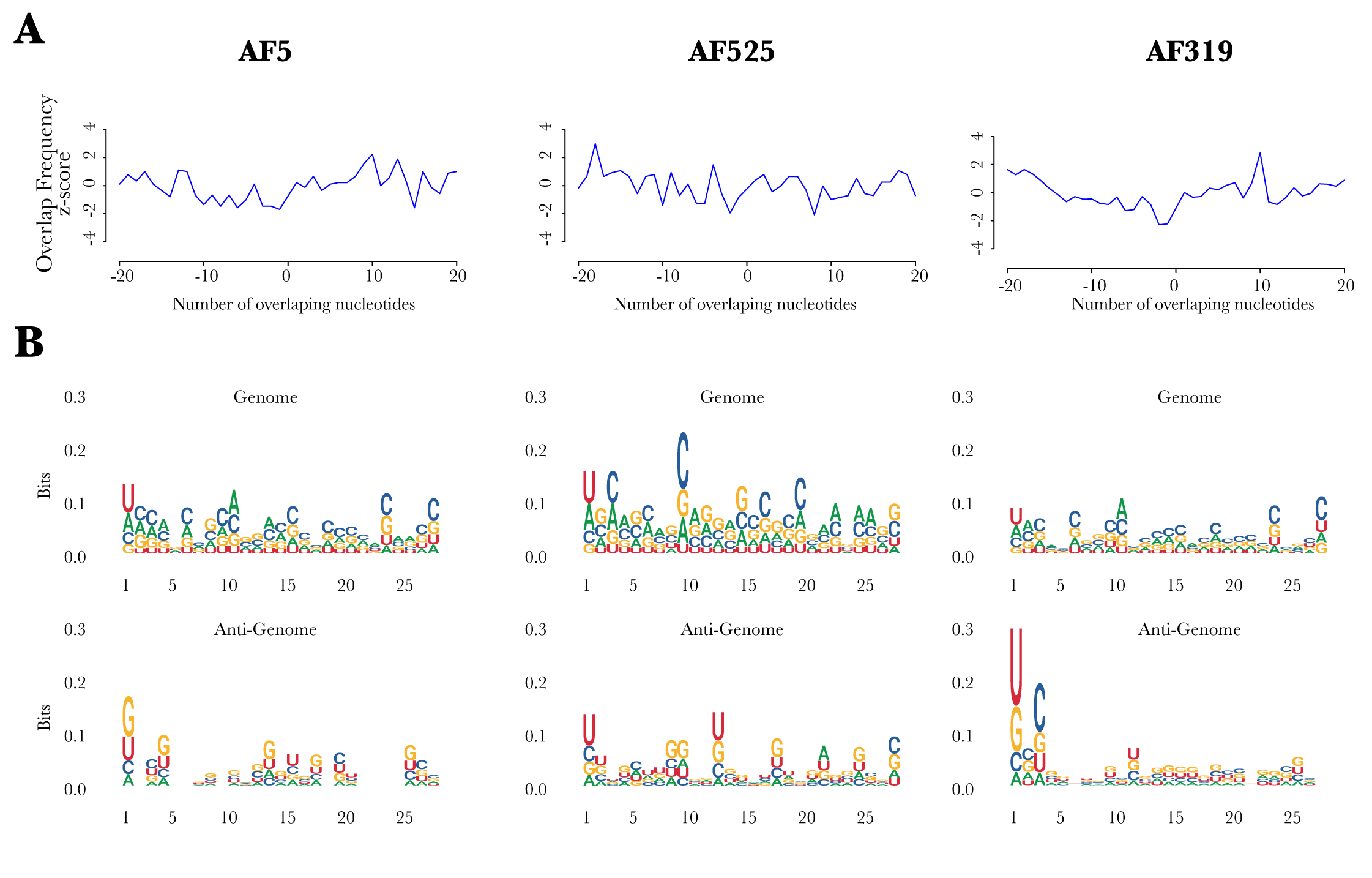

Supplement: FIG S2 [file msphere.01003-21-sf002.tif]

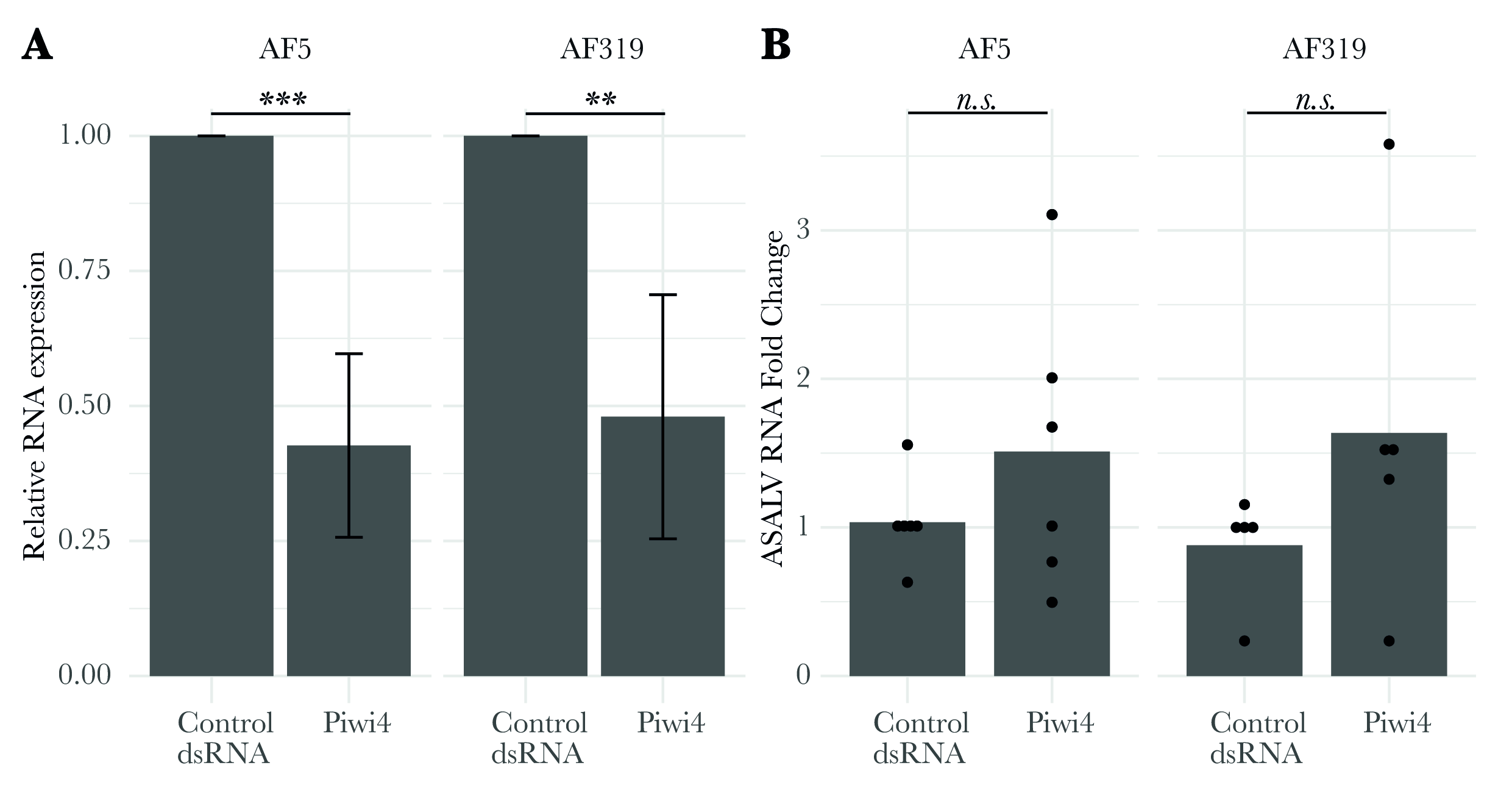

Supplement: FIG S3 [file msphere.01003-21-sf003.tif]

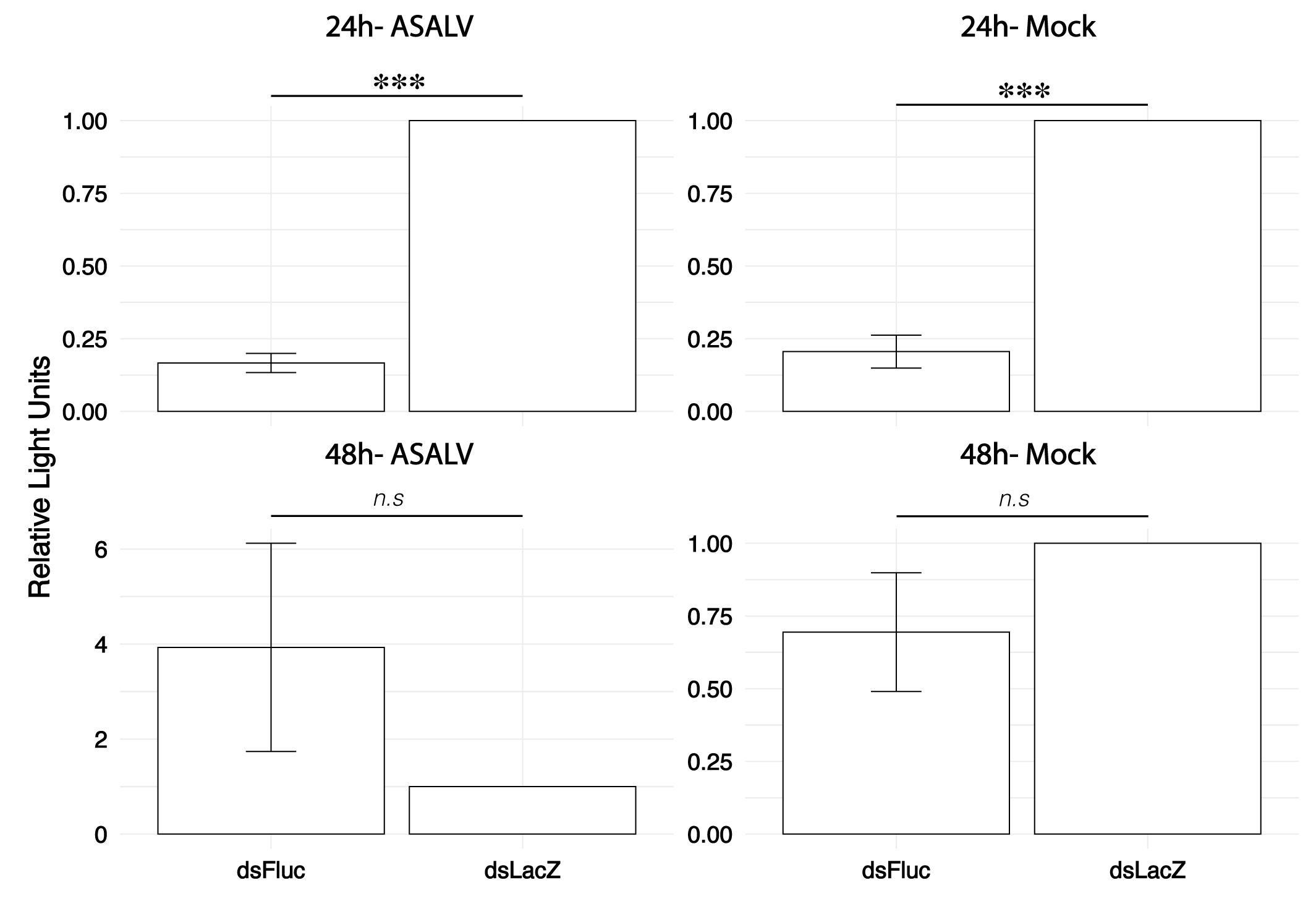

Supplement: FIG S4 [file msphere.01003-21-sf004.tif]
