# Supplementary material for: Antiviral RNAi Response against the Insect-Specific Agua Salud Alphavirus
Source: mSphere. 2022 Feb 16;7(1):e01003-21. doi: 10.1128/msphere.01003-21 (PMC8849343; doi:10.1128/msphere.01003-21)
Supplement: TABLE S1 [file msphere.01003-21-st001.docx]

|  | |  |
| --- | --- | --- |
| **Target** | **Forward/ Reverse primer (5’ -3’)** | **Reference** |
| Ago1 (*Aedes aegypti*) | GTACGATGCGTCGTAAGTAC/GTACTTGTCGAGGAAGTATTTGG | This study |
| Ago2 (*Aedes aegypti*) | GGCTGCTCACCCAATGTATCAAGA/AACCGTTCGTTTTGGCGTTGAT | (1) |
| Piwi4 (*Aedes aegypti*) | CTTCTCCACCACAGCCAATG/GTCCAATCTGCCTGTTCTCCA | (1) |
| Ago3  (*Aedes aegypti*) | TGCTCCAGACGACGGTTTTG/GGGTCAATATAACGGCTCCCAG | This study |
| Piwi5  (*Aedes aegypti*) | CAGTTTTGGAAGACAGAGTTGGA/CCTGCCGTCACTTTGTAATTTTC | This study |
| Piwi6  (*Aedes aegypti*) | TCCGACGTTTTCAAGTTTTGGA / CACTTTACACTGATCCTGCTCG | This study |
| S7  (*Aedes aegypti*) | CCAGGCTATCCTGGAGTTG/ GACGTGCTTGCCGGAGAAC | (1) |
| ASALV | CCGTACTCGAAACAGACATTGC/ TCGTCAACGCCTAGATCCTCTA | (2) |

1. M. Varjak, *et al.*, Characterization of the Zika virus induced small RNA response in Aedes aegypti cells. *PLoS Negl. Trop. Dis.* **11**, 1–18 (2017).

2. K. Hermanns, *et al.*, Agua Salud alphavirus defines a novel lineage of insect-specific alphaviruses discovered in the New World. *J. Gen. Virol.* **101**, 96–104 (2020).
